# Influenza vaccination of cancer patients during PD-1 blockade induces serological protection but may raise the risk for immune-related adverse events

**DOI:** 10.1186/s40425-018-0353-7

**Published:** 2018-05-22

**Authors:** Heinz Läubli, Catharina Balmelli, Lukas Kaufmann, Michal Stanczak, Mohammedyaseen Syedbasha, Dominik Vogt, Astrid Hertig, Beat Müller, Oliver Gautschi, Frank Stenner, Alfred Zippelius, Adrian Egli, Sacha I. Rothschild

**Affiliations:** 1 Department of Internal Medicine, Division of Medical Oncology, University Hospital Basel, Basel, Switzerland; 2 Cancer Immunology, Department of Biomedicine, University of Basel, Basel, Switzerland; 3 Applied Microbiology Research, Department of Biomedicine, University of Basel, Basel, Switzerland; 4 Oncology, Cantonal Hospital Lucerne, Lucerne, Switzerland; 5 Clinical Microbiology, University Hospital Basel, Basel, Switzerland

**Keywords:** Cancer immunotherapy, Immune-related adverse events (irAE), Checkpoint inhibitor, Vaccine response, Influenza vaccine

## Abstract

**Background:**

Immune checkpoint inhibiting antibodies were introduced into routine clinical practice for cancer patients. Checkpoint blockade has led to durable remissions in some patients, but may also induce immune-related adverse events (irAEs). Lung cancer patients show an increased risk for complications, when infected with influenza viruses. Therefore, vaccination is recommended. However, the efficacy and safety of influenza vaccination during checkpoint blockade and its influence on irAEs is unclear. Similarly, the influence of vaccinations on T cell-mediated immune reactions in patients during PD-1 blockade remains poorly defined.

**Methods:**

We vaccinated 23 lung cancer patients and 11 age-matched healthy controls using a trivalent inactivated influenza vaccine to investigate vaccine-induced immunity and safety during checkpoint blockade.

**Results:**

We did not observe significant differences between patients and healthy controls in vaccine-induced antibody titers against all three viral antigens. Influenza vaccination resulted in protective titers in more than 60% of patients/participants. In cancer patients, the post-vaccine frequency of irAEs was 52.2% with a median time to occurrence of 3.2 months after vaccination. Six of 23 patients (26.1%) showed severe grade 3/4 irAEs. This frequency of irAEs might be higher than the rate previously published in the literature and the rate observed in a non-study population at our institution (all grades 25.5%, grade 3/4 9.8%).

**Conclusions:**

Although this is a non-randomized trial with a limited number of patients, the increased rate of immunological toxicity is concerning. This finding should be studied in a larger patient population.

## Background

The development of blocking antibodies that target inhibitory PD-1/PD-L1 or CTLA-4/CD80/CD86 pathways has led to significant improvements in the prognosis of patients suffering from various cancers including metastatic melanoma, non-small cell lung cancer (NSCLC), renal cell carcinoma (RCC), Hodgkin lymphoma, squamous carcinoma of the head and neck (SCCHN) and bladder cancer [1–6]. Checkpoint inhibition has revolutionized cancer therapy of patients with advanced disease by induction of durable remissions and potential cures in some patients [7–9]. PD-1 interactions with its ligands PD-L1 or PD-L2 is an immune checkpoint that is importantly involved in immune homeostasis and prevents extensive tissue destruction by T cells e.g. during viral infections [10], but can also be involved in T cell dysfunction and relapses of viral infections [11, 12]. Checkpoint inhibition with blocking antibodies against PD-1 or PD-L1 augments T-cell immunity [10] – thereby increasing cancer-specific immunity. However, also virus-specific immunity is increased due to blockade of the PD-1 signalling cascade [13, 14]. Treatments with agents targeting the PD-1/PD-L1 axis usually show a good safety profile with a low risk for grade 3 to 5 immune-related adverse events (irAEs) [15–18]. While severe irAEs are an uncommon complication of anti-PD-1/PD-L1 monotherapy, irAEs can be devastating for patients that are affected.

In patients with cancer, infection with influenza viruses is associated with significant morbidity and mortality [19, 20]. Therefore, vaccination as prevention for influenza virus infection is recommended for patients with cancer and in particular for patients that undergo anti-neoplastic therapy [19, 20]. Patients with NSCLC have an additional risk for complications due to concomitant pre-existing lung disorders such as chronic obstructive pulmonary disease (COPD) [21]. Several analyses of the vaccine-induced humoral immune response in patients undergoing classical cytotoxic chemotherapy have been performed [22–26]. In general, studies have shown that concomitant vaccination against seasonal influenza strains is safe in patients undergoing cytotoxic chemotherapy. However, most of these studies showed a reduced efficacy to mount seroprotective post-vaccine antibody titers [22–24]. While the humoral immune response in patients receiving cytotoxic chemotherapy is reduced, the response in patients undergoing checkpoint blockade for cancer is unknown.

This study aimed to determine the quantity and quality of influenza-specific immune responses and the frequency, type and severity of irAEs in cancer patients undergoing immunotherapy with antibodies targeting the PD-1/PD-L1 pathway.

## Methods

### Patients and vaccine

Patients undergoing checkpoint blockade were vaccinated with an inactivated, non-adjuvanted, trivalent influenza subunit vaccination (Agrippal, Novartis) as standard of care. The vaccine contained the following viruses: Influenza/A/H1N1/California/2009, Influenza/A/H3N2/Texas/2012, Influenza/B/Brisbane/2008. The vaccine was given in the recommended standard dose intramuscularly. For an age-matched control cohort, the partners of the patients were vaccinated and included in our analysis as healthy controls. These healthy individuals were not immunosuppressed or received any checkpoint blockade. After the first analysis and the unexpected finding of a high rate of irAEs we retrospectively analyzed the rate of irAEs in an unselected patient population with metastatic NSCLC (*n* = 40) undergoing checkpoint blockade at our institution and not being vaccinated based on their individual decision.

Serum samples and peripheral blood mononuclear cells were collected before vaccination and at days 7, 30 and 60 post-vaccine.

Radiological response was assessed according to Response Evaluation Criteria In Solid Tumors (RECIST) version 1.1. Clinical benefit was defined as patients achieving stable disease (SD) or better for 6 months or more. Adverse events were classified and graded according to the National Cancer Institute Common Terminology Criteria for Adverse Events version 4.0.

Histopathological analysis of tumor tissue was performed at the Institute for Pathology, University of Basel. PD-L1 immunohistochemistry was performed using PD-L1 (E1L3N, Cell Signaling) antibody with a cut-off for positivity at 1% for tumor cells. Gene sequencing was performed by next-generation sequencing using the AmpliSeq Cancer Hotspot Panel version 2 (Thermo Fisher Scientific).

### Measurement of antibody titers

Antibody titers were measured by a hemagglutination inhibition assay according to the WHO-protocol [27]. Briefly, a two-fold serial dilution of serum from patients and healthy controls was added to a fixed concentration of chicken erythrocytes and A/California/7/09 (H1N1), guinea pig erythrocytes and A/Texas/50/2012 (H3N2), and turkey erythrocytes and B/Brisbane/60/08. The respective inhibitory titers of hemagglutination in presence of serum were determined. Seroprotection was defined as a post-vaccine antibody titer at day 30 of ≥1:40. Seroconversion factor (SCF) was derived by dividing the post-vaccine titer at day 30 by the pre-vaccine titer.

### Flow cytometric analysis of lymphocyte subpopulations

Peripheral blood mononuclear cells (PBMCs) were isolated by centrifugation on Ficoll. PBMCs were stained with anti-CD45, anti-CD3, anti-CD4, anti-CD8, anti-CD45RA, anti-CCR7 and anti-CD62L antibodies (all from Biolegend) and analyzed on a LSR II Fortessa (BD Biosciences). Analysis of relative frequencies was done using FlowJo v10 (FlowJo LLC).

### Inflammatory chemokine measurement

Inflammatory chemokines were measured in the serum of patients collected on days 0, 7, 30 and 60 using a flow cytometry based bead assay that allows simultaneous measurement of 13 inflammatory chemokines (Biolegend). Binding of cytokines to the beads was measured on a LSR II Fortessa (BD Biosciences).

### Statistical considerations

Quantitative data was presented as mean plus or minus the standard deviation or standard error of the mean of three separate assays. Student’s t test was used to compare the mean values within the groups, and the Mann-Whitney U test was used to compare data between the two groups. *p* values less than 0.05 were considered statistically significant. Kaplan Meier statistics was used for survival rates. Statistical analysis was performed with the GraphPad Prism Version 7.0 (GraphPad Software, Inc., La Jolla, CA) and IBM SPSS Statistis Version 22 (IBM, Armonk, NY).

## Results

### Patient characteristics

For this observational study, we included 23 patients with solid cancers at two institutions in Switzerland (University Hospital Basel and Cantonal Hospital of Lucerne).

Median time from initiation of PD-1 blocking antibodies to vaccination was 74 days (range, 4–457 days). Patient characteristics are depicted in Table 1. At the time of analysis, 15/23 (65.2%) patients were still alive. 2/23 (8.7%) patients were still undergoing treatment with the immune checkpoint inhibitor. 11/23 (47.8%) patients had a radiological objective response to immune checkpoint inhibition, while another 5/23 (21.7%) patients had disease stabilization (Table 2). Fourteen patients (60.9%) had a clinical benefit of treatment defined as radiographic response or stable disease for at least 6 months. Median overall survival (OS) in the whole cohort for metastatic disease was 73.5 months. In the subgroup of NSCLC patients median OS is not yet reached. After a mean follow-up of 37.5 months, 10 out of 16 NSCLC patients are still alive. No influenza infection was diagnosed in any of the vaccinated patients in our cohort during the influenza season 2015/2016. The retrospective control cohort to compare the frequency of irAEs consisted of 40 patients with metastatic NSCLC treated with PD-1 inhibitors.

**Table 1:** Patient characteristics

Characteristic	Median (range) or number of patients (%)
Age at diagnosis, years	58.7 years (45.6–84.1)
Gender	
-Male	16 (69.6%)
-Female	7 (30.4%)
Cancer type	
-NSCLC	16 (69.6%)
-RCC	4 (17.4%)
-Melanoma	3 (13.0%)
ECOG Performance Status	
-0	5 (21.7%)
-1	12 (52.2%)
-2	6 (26.1%)
Smoking history	
-Current	4 (17.4%)
-Former	8 (34.8%)
-Never	10 (13.0%)
-Unknown	1 (4.4%)
Immune Checkpoint Inhibitor	
-Nivolumab	22 (95.7%)
-Pembrolizumab	1 (4.3%)
Previous lines of therapy	
-0–1	11 (47.8%)
-2–3	7 (30.4%)
-> 3	5 (21.7%)
Molecular aberration^a^	
-KRAS mutation	7 (30.4%)
-BRAF mutation	2 (8.7%)
-EGFR mutation	1 (4.4%)
-NRAS mutation	1 (4.4%)
-TP53	1 (4.4%)
-Wildtype	6 (26.1%)
PD-L1 Expression	
-0%	1 (4.4%)
-1–5%	1 (4.4%)
-5–10%	1 (4.4%)
-10–20%	1 (4.4%)
-100%	1 (4.4%)
-unknown	18 (78.3%)

^a^Analysis by next-generation sequencing (Oncomine solid tumor panel)

**Table 2:** Radiographic and clinical response to immune checkpoint inhibitors

Response	Number of patients (%)
Radiographic response	
-complete response	0
-partial response	11 (47.8%)
-stable disease	5 (21.7%)
-disease progression	7 (30.4%)
Clinical benefit	
-yes	14 (60.9%)
-no	9 (39.1%)

### Humoral response to influenza vaccination

**Fig. 1:**
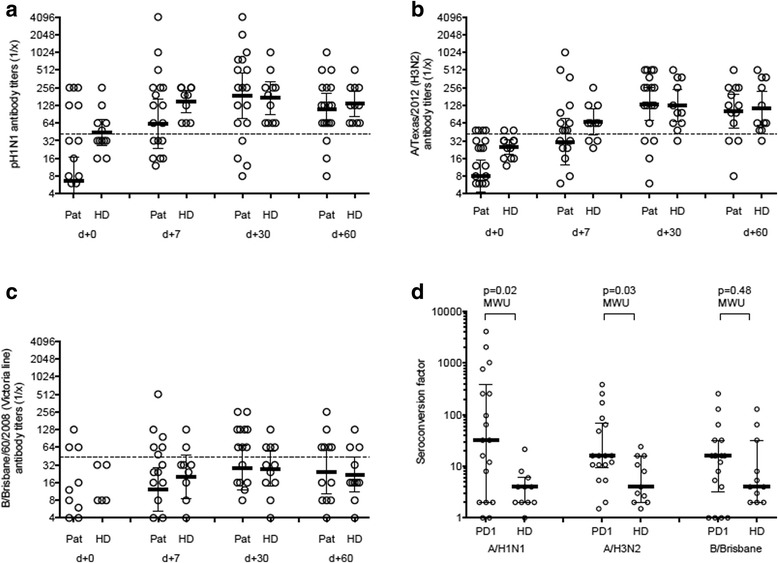
Serological responses to vaccination. Titers from cancer patients undergoing PD-1 blockade (Pat) and healthy age-matched controls (HD) against Influenza A/H1N1 (**a**), Influenza A/H3N2 (**b**), and Influenza B/Brisbane (**c**) after different time points after vaccination. The titers were determined by hemagglutination inhibition assay. The seroconversion factor indicates the ration between post- and pre-vaccine titers for all three antigens at day 30 (**d**). Mann-Whitney U test was used with a significance level of 0.05, two-sided

We compared the antibody titers against three viral antigens within the trivalent vaccine by hemagglutination inhibition assay between cancer patients undergoing PD-1 blockade and healthy age-matched controls (median age (range): 61.7 years (47–86 years)). We did not observe a significant difference in antibody titers against all three viral antigens over time (Fig. 1a-c). The titers against the Influenza B antigen (Victoria lineage) were generally low and both groups (patients and controls) did not reach more than 50% seroprotective titers. The rate of seropositivity was slightly, but non-significantly lower for cancer patients in comparison to healthy controls (A/H1N1: 77.8% vs. 100%; A/H3N2: 77.8% vs. 90.0%) with the exception of B/Brisbane (50% vs. 36.4%). Most interestingly, the seroconversion factor (SCF) was significantly higher in cancer patient in comparison to healthy controls: For A/H1N1, the median was 32 vs. 4 (*p* = 0.02, MWU), for A/H3N2, the median was 16 vs. 4 (*p* = 0.03) (Fig. 1d). This indicated a more potent immune stimulation of cancer patients. Of note, three cancer patients showed a SCF of more than 1000 under PD-1 blockade.

### Changes in inflammatory markers upon vaccination

**Fig. 2:**
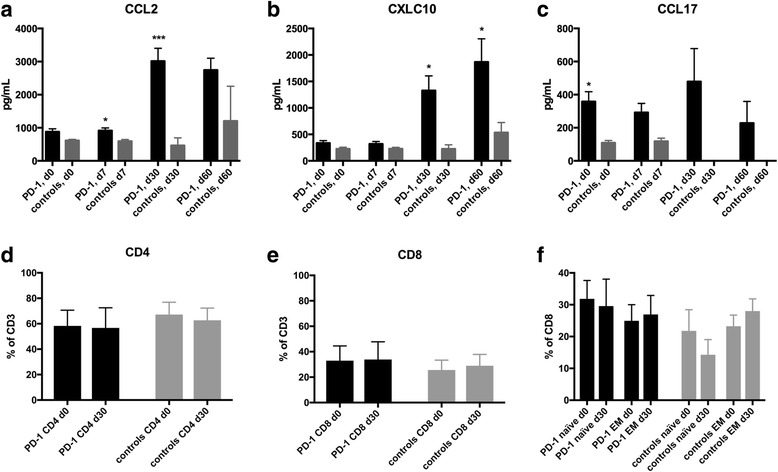
Inflammatory chemokines and lymphocytes in the peripheral blood upon influenza vaccination. (**a**-**c**) Measurement of chemokines before and after vaccination is shown. Chemokines were measured by a multiplex flow cytometry assay. CCL2 (**a**) CXCL10 (**b**) and CCL17 increased over time. (**d)** Measurement of percent of CD4 (**d**), CD8 (**e**) cells were performed by flow cytometry and T cells were defined by gating on living CD45 positive, CD3 positive lymphocytes. (**f**) Determination of naïve (CCR7 positive, CD45RA positive) and effector memory T cells (EM) in peripheral blood upon vaccination. *p < 0.05 by Student’s *t* test, ***p < 0.001 by Student’s *t* test

Since PD-1 blockade might increase immune responses and induce an inflammatory syndrome, we measured inflammatory chemokines in serum of patients under PD-1 blockade to assess the potential induction of an inflammatory syndrome (Fig. 2). Some chemokines including CCL2, CXCL10 and CCL17 were increased compared to age-matched healthy controls (Fig. 2a-c). Over time, there was also a relative increase of CCL2 and CXCL10 in patients undergoing PD-1 blockade (Fig. 2a and b). Median increase of CCL2 was 3.3-fold and of CXCL10 was 5.5-fold. Lactate dehydrogenase (LDH) and C-reactive protein (CRP) levels did not change significantly during the first two weeks after vaccination (median LDH at day 0: 212.6 U/L, day 14: 197.1 U/L; median CRP at day 0: 18.3 mg/L, day 14: 22.8 mg/L). White blood counts including overall numbers of lymphocytes and different non-naïve T-cell subsets were not different between patients undergoing checkpoint blockade and healthy controls (Fig. 2d-f).

### Safety of vaccination

**Fig. 3:**
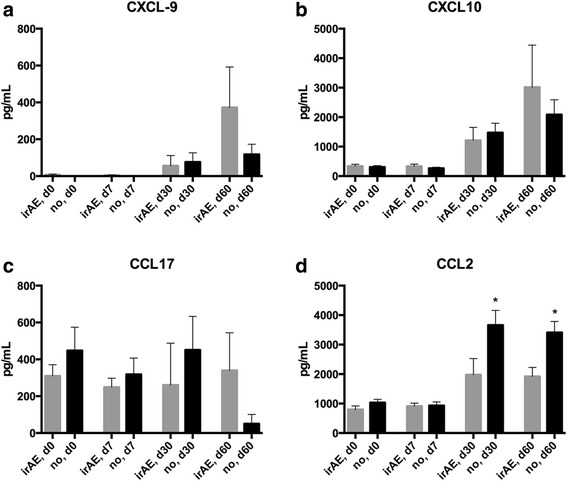
Changes of chemokines in patients with irAEs. Measurement of chemokines after vaccination in patients under PD-1 blockade. A comparison was made between patients that developed sever grade 3/4 irAEs and patients with no side effects of PD-1 blockade. While CXCL9 (**a**), CXCL10 (**b**) and CCL17 (**c**) showed a non-significant trend towards an increased level in patients with irAEs, CCL2 (**d**) was lower in patients who experienced irAEs. *p < 0.05 by Student’s *t* test

The rate of local irritation (all grades) in the area of the vaccine injection in the deltoid muscle was not significantly different to healthy controls (data not shown). While no severe adverse events attributable to influenza vaccination were noted in the patient population during the first 30 days after vaccination, the overall frequency of irAEs was unusually high at 52.2% and 6 out of 23 patients (26.1%) had severe grade 3/4 irAEs (Table 3). The most common side effects (all grades) were rash (outside the vaccination site) (13%), arthritis (13%), and colitis (8.7%) (Table 4). We also observed rare and unusual side effects. Two patient developed encephalitis and one patient a peripheral neuropathy. Patient 010 (male, NSCLC) was operated on a new solitary brain lesion occurring 6.3 months after initiation of nivolumab therapy and 2.0 months after influenza vaccination after having achieved stable disease. Histologically the brain lesion was necrotisizing encephalitis without evidence of tumor cells. Patient 011 (female, NSCLC) was diagnosed with an axonal impairment of the nervus medianus right 6.5 months after treatment start with nivolumab and 5.1 months after influenza vaccination. Radiologically there was no evidence of tumor infiltration, analysis of intraspinal fluid revealed a lymphocytosis without evidence of malignant cells. Anti-GD1a ganglioside antibodies were elevated 2.5-fold. Corticosteroids did not result in symptom improvement. After therapy with intravenous immunoglobulins neuropathy showed complete remission. Median time from initiation of immune checkpoint blockade to the occurrence of the irAE was 6.7 months (range, 1.8–24.6 months). All reported irAEs occurred after influenza vaccination. Median time from vaccination to occurrence of irAEs was 3.2 months (range, 0–10.6 months). In two patients the irAE occurred within the first 30 days after vaccination in all other irAEs occurred with a delay of more than one months after influenza vaccination. This frequency is significantly higher than published safety data of PD-1 checkpoint blockade trials [3, 4, 18] and also significantly higher than in a cohort of 40 metastatic NSCLC patients treated with PD-1 inhibitors at our center (all grades 25.49%, grade 3 or 4 at 9.8%). We also observed a trend for increasing CXCL9, CXCL10, and CCL17 levels in patients, who developed irAEs compared to patients without side effects (Fig. 3a-c). Interestingly, the only significant difference was an increase of CCL2 in patients without irAEs after 30 and 60 days (Fig. 3d).

**Table 3:** Immune-related adverse events

Summary of immune-related adverse events	
Immune-related adverse event	Number of patients (%)
irAE	12 (52.2%)
Grade	
-G1/2	6 (26.1%)
-G3/4	6 (26.1%)
irAE type	
-skin (rash)	3 (13.0%)
-arthritis	3 (13.0%)
-colitis	2 (8.7%)
-encephalitis	2 (8.7%)
-hypothyroidism	1 (4.3%)
-pneumonitis	1 (4.3%)
-neuropathy	1 (4.3%)

**Table 4:** Frequency of specific immune-related adverse events

Immune-related adverse event	G1/2, *n* (%)	G3/4, *n* (%)
Skin (rash)	3 (13.0%)	0
Arthritis	3 (13.0%)	0
Colitis	0	2 (8.7%)
Encephalitis	0	2 (8.7%)
Hypothyroidism	1 (4.3%)	0
Pneumonitis	0	1 (4.3%)
Neuropathy	0	1 (4.3%)

## Discussion

Here we report on the humoral immune response and safety of a trivalent, inactivated, non-adjuvanted influenza vaccine in patients that were treated with a PD-1/PD-L1 blocking agent. The cohort of patients received a seasonal vaccination for the prevention of influenza during the season 2015/2016 in Switzerland. Most of the patients were treated for metastatic NSCLC. In our cohort, the overall seroprotective levels at day 30 were very similar between cancer patients undergoing checkpoint blockade and healthy age-matched controls. However, the seroconversion rate was significantly higher in patients under immune checkpoint blockade, indicating a much more potent immune stimulation in cancer patients compared to healthy individuals and reflecting the relatively low baseline levels in cancer patients. Some patients showed a rapid and massive increase of antibody titers (Fig. 1). The rapid increase and sufficient generation of antibody titers in patients undergoing immunotherapy with PD-1 blocking agents is in clear contrast to previously reported lower antibody titers in cancer patients undergoing cytotoxic chemotherapy [22–26]. These results raise interesting questions regarding the use of PD-1 blockade in a non-systemic application as a vaccine adjuvant. In addition, PD-1 seems to play a role in defective immune responses during viral respiratory infections [11, 12, 28]. In addition, a preclinical analysis in rhesus macaques has shown an enhanced frequency of antiviral T cells upon simian immunodeficiency virus (SIV) vaccination after PD-1 blockade [29] and immune response to herpes virus infection was enhanced upon PD-L1 inhibition in mice [30]. Finally, mice lacking PD-L1 on hematopoietic cells have an increased immune response to an infection with the lymphocytic choriomeningitis virus (LCMV) [31]. Thus, improved responses against viral antigens in patients undergoing PD-1 inhibition are not unexpected. However, in the recently presented retrospective INVIDIa study, a higher incidence of seasonal influenza was reported in patients undergoing immune checkpoint inhibitor therapy [32]. Interestingly, patients receiving vaccination and/or developing influenza infection showed a better overall survival. This finding is in accordance with our NSCLC cohort of 16 patients, in which the median OS is not reached after a follow-up period of more than 3 years (37.5 months).

We observed a significant rate of irAEs following vaccination in the long-term clinical course. The observed frequency was significantly higher than those published as safety data in PD-1 checkpoint blockade trials [15–17]. Patients included in immune checkpoint blockade trials were carefully screened and those at elevated risk for autoimmune disease were excluded. However, safety data from the Italian expanded access program with less stringent inclusion criteria than prospective landmark trials and being similar to daily practice showed comparable rates of irAEs (all grades 29%, grade 3/4 6%) as reported in phase III trials [33]. In an unselected non-study population of 40 metastatic NSCLC patients undergoing immune checkpoint inhibition at our center and not being vaccinated we observed a similar frequency of irAEs compared to a selected trial population (all grades 25.5%, grade 3/4 at 9.8%) and significantly different from the rates observed in vaccinated patients in this study. Severe irAEs were found at a low rate with an average risk in a recent meta-analysis for severe colitis at 1.5%, severe hepatitis/transaminitis at 1.5%, severe dermatitis at 1.1%, hypothyroidism at 0.3% and severe pneumonitis at 1.1% [4]. Other studies and case series demonstrated similar frequencies [15]. Although being a small study, our finding that 52.2% of previously vaccinated patients developed any grade of irAEs and 26.1% had a severe complication of PD-1 blockade raises important concerns about the safety of applying the seasonal influenza vaccination to patients undergoing cancer immunotherapy. It is important to acknowledge that patients responding to immunotherapy were likely overrepresented in our analysis due to a selection bias for patients that were treated for relatively longer times with a PD-1 inhibitor. This bias could also potentially select for patients that have an increased propensity for auto-immune side effects. Combination immunotherapy with blockade of CTLA-4 and PD-1 is approved for metastatic melanoma and is under investigation in several other indications [34]. This combination immunotherapy induces irAEs of any grade in a vast majority of treated patients and Grade 3–4 irAEs in over 50% of patients [34]. It is conceivable that combination immunotherapy has even a higher risk for side effects when combined with vaccinations and safety should be investigated in this patient population. For prophylactic vaccination in patients undergoing immune checkpoint inhibition, safety profiles for different vaccines has to be elucidated. In a retrospective analysis 30 of 108 patients (mainly melanoma) treated with immune checkpoint inhibitors received a total of 53 prophylactic vaccinations (influenza, pneumococcal and others) [35]. The authors did not find a higher rate of all grade irAEs in the vaccinated cohort; G3/4 irAEs were not reported separately.

The exact pathomechanism of irAEs after checkpoint blockade and how the breakdown of tolerance towards self-antigens exactly works in patients with irAEs is not completely understood [36, 37]. Most data are derived from preclinical models and correlative human studies. How the combination of prophylactic vaccination and PD-1 blockade could increase irAEs also remains speculative. The physiological role of the PD-1/PD-L1 pathway is to mediate peripheral tolerance of T cells and inhibition of immune checkpoints could break such tolerance [36, 38]. A recent report in a mouse model has provided evidence that PD-1 blockade together with a viral-based vaccination mediated infiltration of central memory T cells into the tissues, which could also induce auto-reactive immune responses [39]. Studies in patients treated with checkpoint inhibitors show an expansion of auto-reactive T-cell clones upon treatment with checkpoint inhibitors that can also be found in the peripheral blood in patients with irAEs [40, 41]. Identification of T-cell clones by sequencing of the complementarity-determining regions 3 (CDR3) of the T-cell receptor (TCR) beta chain has also shown similar clones to be present in auto-immune lesions in cases of myocarditis compared to those found in the primary lesion or pneumonitis [42, 43]. These findings support a hypothesis that shared antigens in the tumor and the irAE-affected organ can lead to auto-immune disorders by cross-presentation of such shared antigens [36]. Another potential mechanism is the exacerbation of previously subclinical auto-immune syndromes [44, 45]. We have described a case, in which anti-endothelial antibodies were already present before the initiation of PD-1 blockade and upon treatment the patient developed a cerebral vasculitis with necrosis of brain tissue [45]. An additional postulated mechanism of irAE induction is via epitope spreading during checkpoint blockade [36]. It could be speculated that PD-1 blockade together with vaccination – in particular in conjunction with a strong vaccine adjuvant – could boost the breakage of tolerance by enhancing one or several of above mentioned mechanisms associated with irAEs in patients. Moreover, since T cells show cross-reactivity to different antigen-MHC complexes, auto-immunity and irAEs could also be the result of TCR binding degeneracy [46] and cross-reactivity of T cells stimulated by the protein contained in the influenza vaccine to self-peptide-MHC complexes.

Therapeutic vaccination for cancer is currently tested in many clinical trials together with immune checkpoint inhibitors [9, 47]. This is based on preclinical models that have shown clear synergy between checkpoint blockade and vaccination [48–51]. Current strategies involve therapeutic vaccination with tumor epitopes – most often neoantigens – together with PD-1 or PD-L1 blocking antibodies [9, 52–54]. Our findings suggest that combination of therapeutic vaccination with checkpoint blockade could not only increase anti-tumor efficacy but also the rate of irAEs. Ongoing trials will provide more information on the toxicity of vaccine combinations with immune checkpoint blockade.

This study has clear limitations and further investigations are warranted. The small number of patients analyzed precludes a definitive statement on the safety of influenza vaccination in patients undergoing cancer immunotherapy. A larger cohort needs to be analyzed to advise for or against vaccination of patients that recently received therapy targeting the PD-1/PD-L1 axis. Moreover, predictions for newer therapeutic strategies that include immunotherapy cannot be made on the basis of this analysis. In particular, patients receiving combination immunotherapy including the combination of CTLA-4 and PD-1 inhibitors were not analyzed and the risk for adverse events should be investigated separately in this patient population. Although the observed rate of irAEs in our cohort is of concern, we believe that there is particular concern for patients with lung cancer under immunotherapy for severe complications of an influenza infection including pneumonia and respiratory failure because of concomitant structural lung disorders [55]. Some of these patients had prior resection of lung lobes or even a pneumonectomy and have therefore limited reserves due to impaired lung capacity. Moreover, the unexpectedly long survival of NSCLC patients in this cohort warrants further investigations in prospective clinical trials to understand if prophylactic vaccination may improve the outcome of cancer patients undergoing immune checkpoint blockade. When weighting benefit and potential risk of seasonal influenza vaccination for patients undergoing single-agent PD-1 or PD-L1 blockade – in particular those with lung cancer – we currently advice to make an individual decision against or for an influenza vaccination until results from larger cohorts are available.

## Conclusions

This is the first analysis demonstrating adequate humoral immune response of a trivalent, inactivated, non-adjuvanted influenza vaccine in patients that were treated with a PD-1/PD-L1 blocking agent. However, there might be the potential of a higher rate of irAEs induced by immune checkpoint inhibitors in patients undergoing influenza vaccination.

## Abbrevations

CCL2: C-C motif chemokine ligand
CCR-2: C-C chemokine receptor type 2
CDR3: Complementarity-determining regions 3
CTLA4: Cytotoxic T-lymphocyte-associated protein 4
irAE: Immune-related adverse event
MHC: Major histocompatibility complex
NSCLC: Non-small cell lung cancer
PD1: Programmed cell death protein 1
PD-L1: Programmed cell death protein ligand 1
RCC: Renal cell carcinoma
SCCHN: Squamous carcinoma of the head and neck
TCR: T-cell receptor
